# Supplementary material for: Inefficient nitrogen transport to the lower mantle by sediment subduction
Source: Nat Commun. 2024 Aug 14;15:6998. doi: 10.1038/s41467-024-51524-1 (PMC11324759; doi:10.1038/s41467-024-51524-1)
Supplement: Supplementary file 1 — Supplementary information [file 41467_2024_51524_MOESM1_ESM.pdf]

Supplementary Information for

Inefficient nitrogen transport to the lower mantle by sediment subduction

Weihua Huang[1], Yan Yang[1,*], Yuan Li[2,3], Zheng Xu[2], Shuiyuan Yang[4], Shengbin Guo[4], Qunke Xia[1]

[1] Key Laboratory of Geoscience Big Data and Deep Resource of Zhejiang Province, School of Earth Sciences, Zhejiang University, Hangzhou 310027, China

[2] State Key Laboratory of Isotope Geochemistry, Guangzhou Institute of Geochemistry, Chinese Academy of Sciences, Guangzhou 510640, China

[3] Bayerisches Geoinstitut, Universität Bayreuth, Bayreuth 95440, Germany

[4] State Key Laboratory of Geological Processes and Mineral Resources, China University of Geosciences, Wuhan 430074, China

[*] To whom correspondence should be addressed. Email: yanyang2005@zju.edu.cn

Content of this file

Supplementary Texts 1-6

Supplementary Figures 1-7

Supplementary Tables 1-2

Supplementary Text 1: Phase compositions of the run products

The major elements and nitrogen contents of the minerals in all the experimental runs are provided in Supplementary Data 1. Except for garnet, the compositions of all minerals are homogeneous.

For experiments using NH_3 solutions, garnet zones with Fe-rich cores always occur at temperatures lower than 1100 °C. This may be caused by the rapid crystallization of garnet from the starting material at low temperatures [1]. In contrast to cores rich in almandine components, rims contain more pyrope and grossular components. Thus, the compositions of the rim and core were considered two separate phases during the mass balance calculations. In contrast, garnets formed in the NH_4NO_3-bearing run have homogeneous compositions, but the Fe content is much lower than that in other runs (Supplementary Fig. 1a). The measurement of nitrogen was only carried out on garnet without obvious zonal texture to prevent potential effects of inhomogeneous compositions. However, no nitrogen signal was detected.

Clinopyroxene in the NH_3-bearing runs has a composition similar to jadeite ($NaAlSi_2O_6$), with a minor diopside ($CaMgSi_2O_6$) component. The jadeite component increases with pressure, which corresponds well with the trend observed in previous studies (Supplementary Fig. 1b). In contrast to the negligible Fe content in clinopyroxene of the NH_3-bearing runs, clinopyroxene in the NH_4NO_3-bearing run contains 6.07 wt% FeO^{tot} (all Fe is considered FeO). The obviously lower Al content indicates that Fe is incorporated as aegirine ($NaFe^{3+}Si_2O_6$) component (Supplementary

Fig. 1b). The nitrogen content was measured only for some large clinopyroxene grains in the high-temperature runs. No nitrogen was detected in clinopyroxene by EPMA, with a detection limit of ~300 ppm. This result is consistent with the nitrogen content of only 90±70 ppm in Al-clinopyroxene synthesized at 11.5 GPa [2]. Raman spectroscopy also did not reveal any nitrogen signals in clinopyroxene (Supplementary Fig. 2a).

Phengite has a Si content of 3.76 atoms per formula unit (apfu) with K^+ as a major interlayer ion, and the content of Na is close to zero. It contains 4891±975 ppm nitrogen. An asymmetric peak near ~3210 cm^{-1} in the Raman spectrum suggests ammonium vibration modes (Supplementary Fig. 2b). In addition, the number of atoms per formula unit (apfu) of $K^+ + Na^+ + NH_4^+$ in phengite approximately equals 1 when all nitrogen is considered to be ammonium. These findings suggest that nitrogen is incorporated into phengite as ammonium and that the vacancy in phengite is negligible.

In all the experimental runs, K-hollandite has major element compositions similar to those of $KAlSi_3O_8$ but has a lower alkali cation content. The contents of the other elements are close to zero. EPMA measurements revealed that the nitrogen contents in K-hollandite of the NH_3-bearing runs varied from 1020±399 ppm to 8919±1164 ppm. Raman spectroscopy revealed peaks in the range of 2700-3400 cm^{-1} (Supplementary Fig. 2c), which correspond to the stretching vibrations of ammonium [3]. The ratio of the intensity of the strongest ammonium peak at 3135 cm^{-1} to the sum of the intensities of the peaks at 214 cm^{-1} and 763 cm^{-1} strongly correlates with the nitrogen content measured by EPMA (Supplementary Fig. 2d). In addition, the nitrogen content strongly negatively correlates with the K_2O content (Supplementary Fig. 1c). These findings

suggest that nitrogen is mainly incorporated as ammonium in K-hollandite. For K-hollandite in the NH_4NO_3-bearing run, no nitrogen was detected by EPMA or Raman spectroscopy (Supplementary Fig. 2c), and $K^+ + Na^+$ was calculated to be 1 apfu, so the nitrogen content is considered to be zero.

Kyanite and topaz-OH in the NH_3-bearing runs display nearly stoichiometric compositions, and the Fe contents are negligible. In contrast, approximately 4.61 wt% FeO^{tot} was detected in the kyanite in the NH_4NO_3-bearing run. The incorporation of Fe in kyanite is strongly controlled by oxygen fugacity, and the Fe content in kyanite correlates well with the ratio of Fe^{3+} to Fe^{2+} in the whole rock [4]. Therefore, the high Fe content in kyanite implies the high oxygen fugacity of the NH_4NO_3-bearing run.

Stishovite grains are often tiny and needle-like in shape, making analysis challenging. Therefore, the compositions of stishovite were only successfully quantified in the 10 GPa-1000 °C and 10 GPa-1100 °C runs. Apart from Si and Al, the contents of other elements are negligible. Approximately 0.78 wt% and 1.14 wt% Al_2O_3 are incorporated in stishovite at 1000 °C and 1100 °C, respectively. The composition of stishovite in the 10 GPa-1100 °C run was used in other runs for mass balance calculations. Due to the small grain sizes, nitrogen analysis was not carried out.

Small amounts of Ti oxide containing minor Al_2O_3 is present in some NH_3-bearing runs. In the NH_4NO_3-bearing run, the Fe oxide and Fe-Ti phase are observed. Fe oxide contains minor TiO_2 and Al_2O_3 and was identified as hematite by Raman spectroscopy. The Fe-Ti phase contains ~48.5 wt% TiO_2 and ~33.5 wt% FeO^{tot}, with a total value of

~88.4 wt%. This result is consistent with the Fe-Ti oxyhydroxide observed in a previous study [5].

Quench materials are observed in some high-temperature runs (1000-1100 °C), suggesting that major elements become more soluble in fluid at higher temperatures. Semiquantitative analyses show that the compositions of the quench materials are heterogeneous and that the materials are mainly rich in Si, K, and Al.

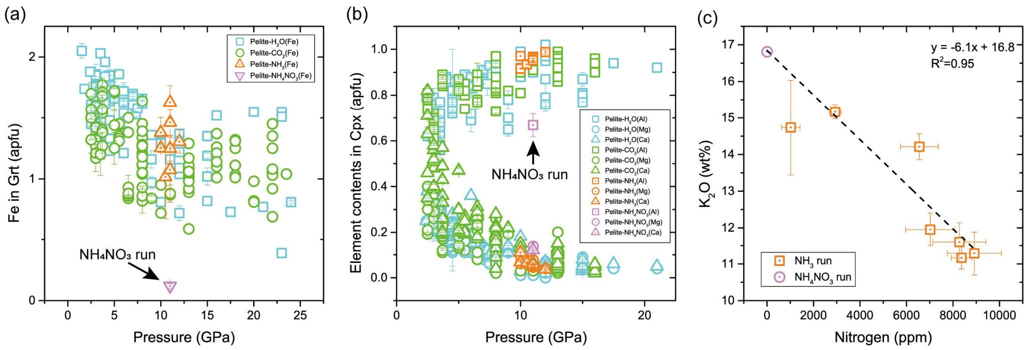

Supplementary Fig. 1 Chemical compositions of garnet, clinopyroxene, and K-hollandite. (a) Correlation between the Fe content in garnet and pressure. The Fe content of the garnet core is not included. (b) Correlations between Al, Mg, and Ca contents in clinopyroxene and pressure. The data for the pelite-H_2O system (cyan) [6–9] and the pelite-CO_2 system (green) [8,10–12] are from Refs. The pelite-NH_3 (orange) and pelite-NH_4NO_3 (magenta) systems are from the NH_3-bearing runs and NH_4NO_3-bearing run in this study (Supplementary Data 1). Error bars represent the standard deviation based on Monte Carlo simulations that compound uncertainties for oxides (wt%). (c) Correlation between nitrogen content and K_2O in K-hollandite in this study. Error bars represent the standard deviation.

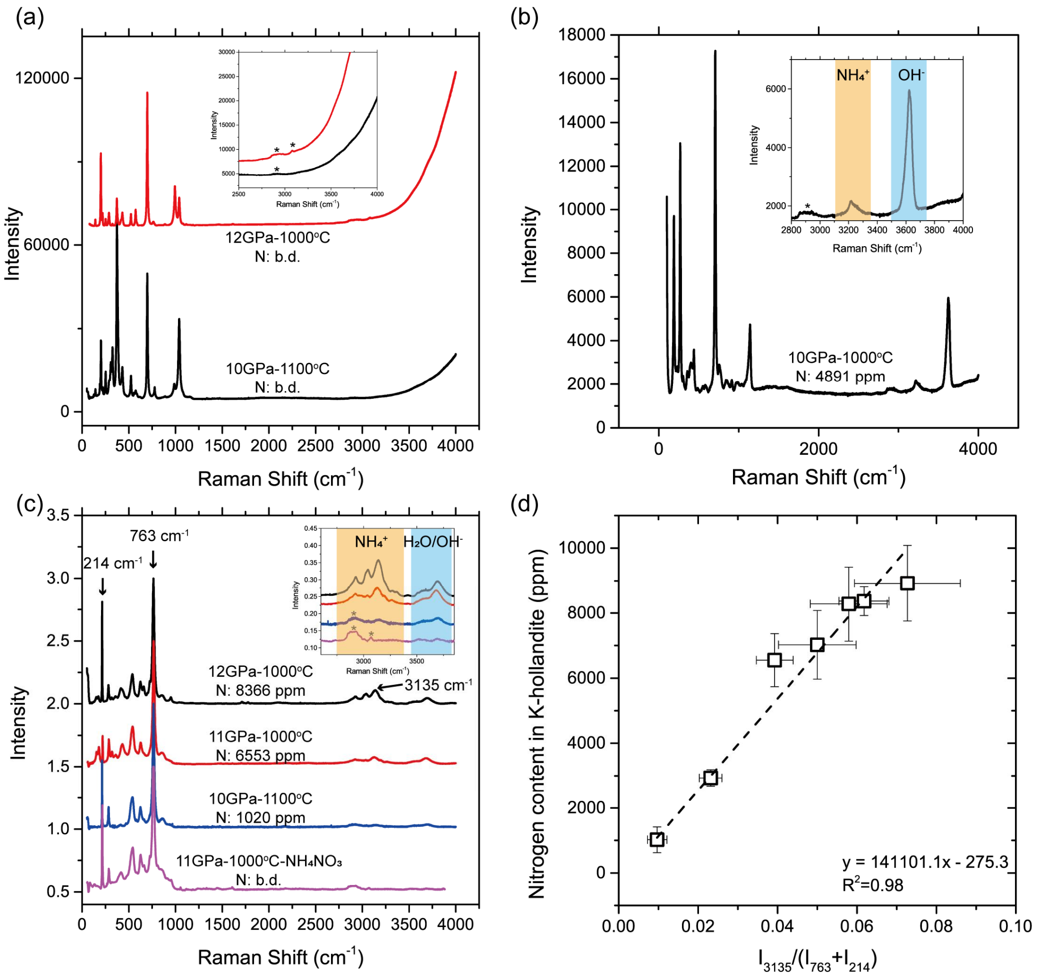

Supplementary Fig. 2 Nitrogen measurements using Raman spectroscopy. Raman spectra of (a) clinopyroxene, (b) phengite, and (c) K-hollandite. Asterisks indicate the peaks influenced by the contamination of resin. The asymmetric peak near ~3620 cm^{-1} for phengite represents the OH vibration. Peaks in the range from 3400 to 3800 cm^{-1} for K-hollandite may also be due to the OH vibrations. (d) Correlation between the nitrogen content in K-hollandite measured by EPMA and the intensity ratio of the 3135 cm^{-1} peak to the sum of the 214 cm^{-1} and 763 cm^{-1} peaks. Error bars represent the standard deviation. Source data are provided in Supplementary Data 1.

Supplementary Text 2: Mass balance calculations

Using the major element compositions of the starting materials and run products, the phase proportions were calculated based on mass balance using a least-squares procedure. The Monte Carlo approach was used to evaluate the errors induced by the measurement uncertainties for the major element.

In some runs, phengite and K-hollandite contain high contents of ammonium which substitutes for potassium, while the K_2O contents in other phases are close to zero. Thus, the fluid should include at least the same quantity of potassium as ammonium in phengite and K-hollandite. As a result, the abundances of phengite and K-hollandite are overestimated when the effect of ammonium is ignored. Here, the effect of ammonium was corrected by the following method. First, the mineral proportions were determined without considering the ammonium concentration in the minerals. Combining the calculated modes of phengite and K-hollandite with the ammonium contents in these minerals, the amount of ammonium in the solid phase was obtained. This value is equal to the lower limit of the amount of potassium in fluid. The mass balance calculations were carried out again, but this time the K_2O content utilized was the K_2O content in the starting material minus the K_2O content in the fluid phase. This process was iterated 20 times to ensure that the mineral proportions did not obviously change any more, and the final results are the average results of the last 10 cycles. The phase proportions are provided in Supplementary Data 1.

Supplementary Text 3: Estimating the nitrogen transport efficiency passing through the sub-arc depth at the Central American margin

The nitrogen transport efficiency passing through the sub-arc depth in the Central American (CA) margin is calculated based on the mass balance between the influx and outflux of sedimentary nitrogen. The input nitrogen flux from subducted sediments is ~1.7×10^7 kg/y [13,14] and the average output nitrogen fluxes from arcs are ~8.2×10^6 kg/y [15] and ~2.0×10^7 kg/y [16]. The degassed N_2 from the arcs also includes nitrogen lost from

altered oceanic crust (AOC), which consists of upper basaltic crust and lower gabbroic crust with average input nitrogen fluxes of ~0.8×10^7 kg/y and ~1.3×10^7 kg/y, respectively [14]. The nitrogen content of the altered basalts, altered gabbroic rocks and their eclogite-facies metamorphosed equivalents in the warm slabs are compared, revealing that the nitrogen loss contributed by the upper basaltic crust may be negligible, and ~48% of the nitrogen in the lower gabbroic crust may be lost [17,18]. Therefore, the sediment-derived N_2 output CA arc is calculated to be ~ 0.2-1.3×10^7 kg/y, implying that the recycling efficiency is ~24-89% for a warm subduction zone of the Central American margin.

Supplementary Text 4: Uncertainty caused by sample loading

For the NH_3-bearing runs, a NH_3 (25 wt%)-H_2O solution was used as the water and nitrogen source. The powders were loaded into the Au capsule, and the capsule was welded with a small hole left. The size of the hole barely allows the needle of microsyringe passing through. The NH_3 solution was loaded into the Au capsule through this hole by a microsyringe. The added volume of NH_3 solution was calculated by the ideal mass of NH_3 solution and its density (0.9). The left hole was rapidly closed and welded shut while being cooled in cold water. The gas-tightness of the final Au capsule was verified by heating it at 110 °C for at least one hour. This method could minimize the loss of fluid during the loading process. The difference between the capsule weight before the addition of NH_3 solution and that after the gas-tightness test is considered as the mass of NH_3 solution in the capsule. By comparison with the ideal mass of NH_3 solution that should be loaded in, we can estimate the maximum

uncertainty induced by the loading and welding processes. The fluid retention in the capsule ranges from ~77 to 94%. This indicates that ~6.2-7.5 wt% fluid has been added to the system. However, the concentration of NH_3 in fluid should not change greatly due to the consistent P-T dependence of the nitrogen content in K-hollandite, despite variations in fluid retention among different runs. For example, the 10 GPa-1100 °C run (NH-2) with a fluid retention of 94% has the lowest nitrogen content in K-hollandite, while the 12 GPa-1000 °C run (NH-8) with a fluid retention of 77% has the second highest nitrogen content in K-hollandite. Therefore, the fluid mass fraction of each run was concurrently corrected for both water and NH_3 during the calculation of partition coefficients (Supplementary Table 1).

For the NH_4NO_3-bearing run, 7.49 mg of starting material powder, 0.39 mg of NH_4NO_3 powder, and 0.32 mg of water were added to the Au capsule, which should introduce ~1.66 wt% N and 6 wt% H_2O into the system. Then the capsule was welded shut while being cooled in cold water. The difference between the capsule weight before welding and after the gas-tightness test was 0.06 mg, indicating that at least ~81 wt% water was added to the capsule. Therefore, the actual mass of fluid in the capsule should be ~1.68 wt% N and ~5.4 wt% H_2O.

Supplementary Table 1 Detailed fluid retention for each NH_3-bearing run.

Runs	Fluid retention (%)	Corrected H_2O (wt%)	Corrected NH_3 (wt%)	Silicate and volatile mass ratios
NH-1	78	4.7	1.6	14.9
NH-2	94	5.7	1.9	12.2
NH-3	90	5.4	1.8	12.9
NH-4	77	4.6	1.5	15.4
NH-5	85	5.1	1.7	13.7
NH-6	78	4.7	1.6	14.9
NH-7	77	4.6	1.5	15.4
NH-8	77	4.6	1.5	15.4

Supplementary Text 5: N Kα peak positions of BN and K-hollandite

The N Kα peak of the BN standard was directly scanned. To determine the position of the N Kα peak of the K-hollandite samples, we measured the intensities at the discrete wavenumbers using long counting times. The backgrounds were corrected using an exponential model (Supplementary Fig. 3ab), and the peak positions of the N Kα peaks are obtained through Gaussian + Lorentzian peak-fitting (Supplementary Fig. 3cd). We repeated the background-correction and peak-fitting for multiple times, and each time different data points were selected to fit the exponential baseline. This procedure allowed us to obtain multiple corrected N Kα peaks and their corresponding peak positions. The average value and its standard deviation of the peak positions were considered as the final peak position and its uncertainty, respectively. The N Kα peak of BN was located at 147.54 ± 0.003 mm, while the N Kα peak of K-hollandite was at 147.21 ± 0.01 mm.

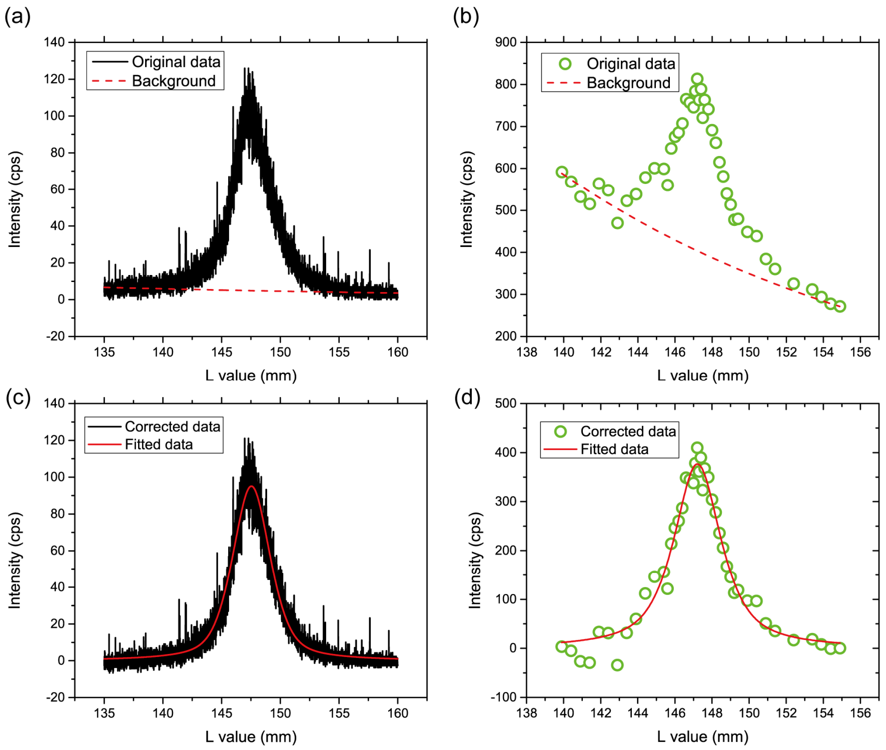

Supplementary Fig. 3 Comparison of N Kα peaks between BN standard and K-hollandite. Baseline correction using an exponential model for the N Kα peak of (a) BN and (b) K-hollandite (NH-8). Peak fitting of the corrected N Kα peak of (c) BN and (d) K-hollandite with a Gaussian + Lorentzian function. The N Kα peak positions of BN and K-hollandite are 147.54±0.003 mm and 147.21±0.01 mm, respectively. Source data are provided in Supplementary Data 1.

Supplementary Text 6: Principles of algorithms

The Random Forest [19], Extremely Randomized Trees (Extra-Trees) [20], and Extreme Gradient Boosting (XGBoost) [21] algorithms were used in this study. The excellent performance of these algorithms in solving regression problems in Earth science with small datasets has been demonstrated by several previous works [22–25]. The Random Forest is an ensemble learning algorithm that combines the bagging algorithm [26] and random feature selection [27]. It consists of many decision trees, and each tree is trained by a subset that is randomly selected with replacement from the whole dataset. When each tree splits at a node, the feature that most satisfies the indicators of feature

selection is chosen from a randomly selected feature subset rather than from the entire feature set. The final prediction is the average value of the results of these decision trees. The Extra-Trees algorithm is similar to the Random Forest algorithm. The main differences from the Random Forest are that the Extra Trees use the entire dataset to train each decision tree, and the features for splitting are selected completely at random. XGBoost is an advanced implementation of Gradient Boosting Decision Tree (GBDT) algorithm [28]. GBDT starts with a decision tree that makes predictions. New trees are continuously created to predict the residuals of prior models to improve the model. The final model aggregates the results of each step, and thus, a strong learner is achieved.

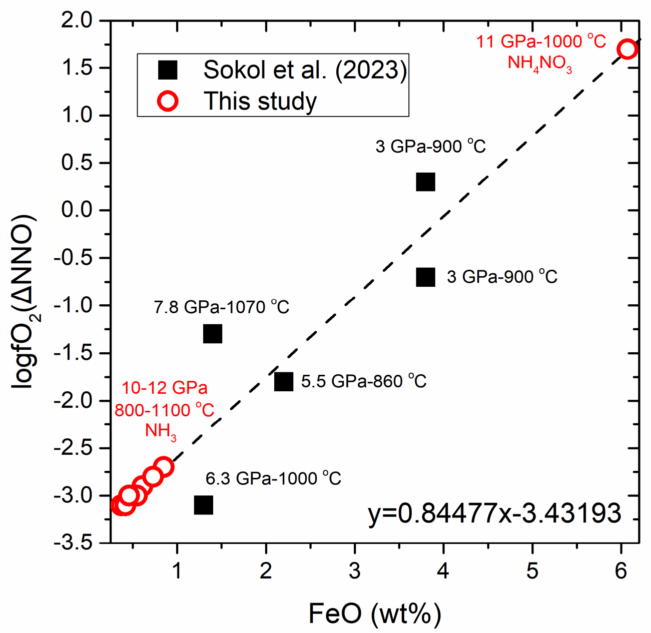

Supplementary Fig. 4 Correlation between the FeO (wt%) in clinopyroxene and the oxygen fugacity. Data from Ref [32].

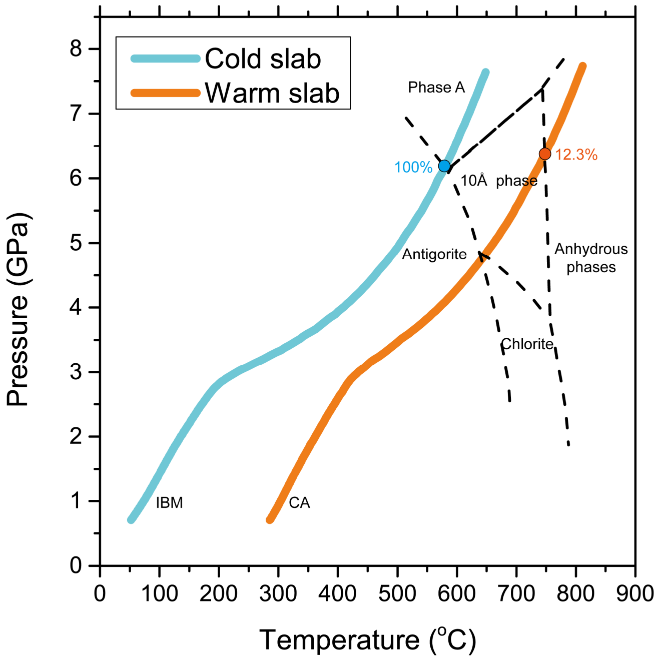

Supplementary Fig. 5 Illustration of nitrogen preservation in phengite considering serpentinite dehydration. P-T paths of the slab Moho (D80 model [29]). The phase relations of water saturated peridotite [30] are displayed by black dashed lines. In the cold slab (Izu-Bonin-Mariana, cyan line), antigorite directly transfers to Phase A, and no fluid will be released up to the depth of the mantle transition zone [30]. Therefore, 100% of the nitrogen can be preserved in phengite and carried to the phengite to K-hollandite transition depth. In the warm slab (Central American, orange line), antigorite completely dehydrates and significant fluid is produced, which causes nitrogen loss from phengite. The nitrogen preservation of phengite in the warm slab is estimated based on the assumption that all fluid lost from a 2 km thick serpentinized slab mantle with 2 wt% H_2O is added to the sediment slab. The mass of phengite is calculated by the mass of sediments and a phengite abundance of 29.9 wt% (this work). The mass ratio of phengite to fluid is ~1.1. The P-T dependence of $D_N^{Phe/Fluid}$ has been poorly constrained, so the average value (0.12) of $D_N^{Phe/Fluid}$ at 5.5 and 6.3 GPa from Refs. [31,32] is applied. Finally, the nitrogen preservation in phengite in the warm slab is ~12.3% considering the fluid from the serpentinized mantle slab. Source data are provided in Supplementary Data 2.

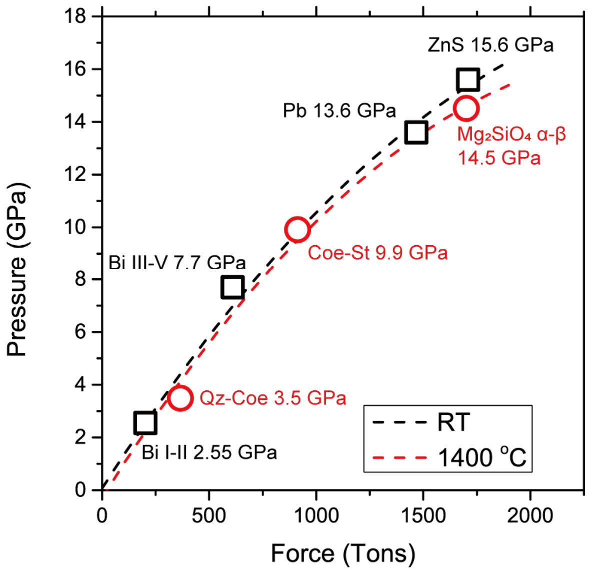

Supplementary Fig. 6 Pressure calibration using 18/11 assemblies in the 2500-ton Cubic-style multi-anvil apparatus UHP-2500 at the Guangzhou Institute of Geochemistry, Chinese Academy of Sciences. The pressure-force relations of the best fit functions are $P = -2.164300\times10^{-6} F^2 + 1.264096\times10^{-2} F + 7.852733\times10^{-2}$ for 25 °C (black curve), and $P = -2.504000\times10^{-6} F^2 + 1.301033\times10^{-2} F - 2.898133\times10^{-1}$ for 1400 °C (red curve), where P is pressure (GPa) and F is force (tons).

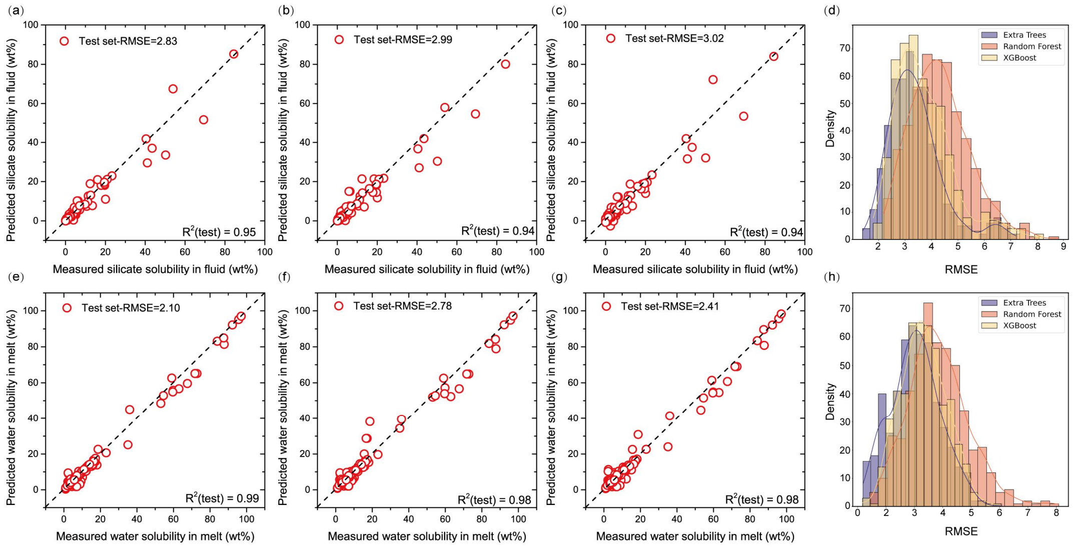

Supplementary Fig. 7 Performances of different machine learning models. Correlations between measured silicate solubility in fluid and that predicted by (a) Extra-Trees, (b) Random Forest, and (c) XGBoost regressions. (d) The distributions of RMSEs in 500 validation sets for the fluid models. Correlations between measured water solubility in melt and that predicted by (e) Extra-Trees, (f) Random Forest, and (g) XGBoost regressions. (h) The distributions of RMSEs in 500 validation sets for the melt models. Source data are provided in Supplementary Data 3.

Supplementary Table 2 Performances of machine learning models.

	Extra Trees	Random Forest	XGBoost
**Performance of fluid models**			
RMSE (validation)	3.39 (101)	4.32 (112)	3.72 (111)
R^2 (validation)	0.93 (4)	0.88 (5)	0.91 (5)
RMSE (test)	2.83	2.99	3.02
R^2 (test)	0.95	0.94	0.94
**Performance of melt models**			
RMSE (validation)	3.04 (87)	3.84 (106)	3.32 (80)
R^2 (validation)	0.98 (2)	0.96 (2)	0.97 (2)
RMSE (test)	2.10	2.78	2.41
R^2 (test)	0.99	0.98	0.98

References

1. Schmidt, M. W., Vielzeuf, D. & Auzanneau, E. Melting and dissolution of subducting crust at high pressures: the key role of white mica. *Earth Planet. Sci. Lett.* **228**, 65–84 (2004).
2. Watenphul, A., Wunder, B., Wirth, R. & Heinrich, W. Ammonium-bearing clinopyroxene: A potential nitrogen reservoir in the Earth's mantle. *Chem. Geol.* **270**, 240–248 (2010).
3. Watenphul, A., Wunder, B. & Heinrich, W. High-pressure ammonium-bearing silicates: Implications for nitrogen and hydrogen storage in the Earth's mantle. *Am. Mineral.* **94**, 283–292 (2009).
4. Müller, A., Van Den Kerkhof, A. M., Selbekk, R. S. & Broekmans, M. A. T. M. Trace element composition and cathodoluminescence of kyanite and its petrogenetic implications. *Contrib. Mineral. Petrol.* **171**, 70 (2016).
5. Nishihara, Y. & Matsukage, K. N. Iron-titanium oxyhydroxides as water carriers in the Earth's deep mantle. *Am. Mineral.* **101**, 919–927 (2016).
6. Hermann, J. & Spandler, C. J. Sediment Melts at Sub-arc Depths: an Experimental Study. *J. Petrol.* **49**, 717–740 (2008).
7. Irifune, T., Ringwood, A. E. & Hibberson, W. O. Subduction of continental crust and terrigenous and pelagic sediments: an experimental study. *Earth Planet. Sci. Lett.* **126**, 351–368 (1994).
8. Mann, U. & Schmidt, M. W. Melting of pelitic sediments at subarc depths: 1. Flux vs. fluid-absent melting and a parameterization of melt productivity. *Chem. Geol.*

404, 150–167 (2015).

9. Ono, S. Stability limits of hydrous minerals in sediment and mid-ocean ridge basalt compositions: Implications for water transport in subduction zones. *J. Geophys. Res.: Solid Earth* **103**, 18253–18267 (1998).
10. Domanik, K. J. & Holloway, J. R. Experimental synthesis and phase relations of phengitic muscovite from 6.5 to 11 GPa in a calcareous metapelite from the Dabie Mountains, China. *Lithos* **52**, 51–77 (2000).
11. Grassi, D. & Schmidt, M. W. The Melting of Carbonated Pelites from 70 to 700 km Depth. *J. Petrol.* **52**, 765–789 (2011).
12. Grassi, D. & Schmidt, M. W. Melting of carbonated pelites at 8–13 GPa: generating K-rich carbonatites for mantle metasomatism. *Contrib. Mineral. Petrol.* **162**, 169–191 (2011).
13. Li, L. & Bebout, G. E. Carbon and nitrogen geochemistry of sediments in the Central American convergent margin: Insights regarding subduction input fluxes, diagenesis, and paleoproductivity. *J. Geophys. Res.: Solid Earth* **110**, (2005).
14. Li, K. & Li, L. Nitrogen enrichments in sheeted dikes and gabbros from DSDP/ODP/IODP Hole 504B and 1256D: Insights into nitrogen recycling in Central America and global subduction zones. *Geochim. Cosmochim. Acta* **335**, 197–210 (2022).
15. Hilton, D. R., Fischer, T. P. & Marty, B. Noble Gases and Volatile Recycling at Subduction Zones. *Rev. Mineral. Geochem.* **47**, 319–370 (2002).
16. Labidi, J. *et al.* Recycling of nitrogen and light noble gases in the Central American

subduction zone: Constraints from $^{15}N^{15}N$. *Earth Planet. Sci. Lett.* **571**, 117112 (2021).

17. Li, K. & Li, L. Nitrogen enrichment in the altered upper oceanic crust: A new perspective on constraining the global subducting nitrogen budget and implications for subduction-zone nitrogen recycling. *Earth Planet. Sci. Lett.* **602**, 117960 (2023).
18. Li, K. & Li, L. Alteration enrichment of nitrogen in the gabbroic oceanic crust: Implications for global subducting nitrogen budget and subduction-zone nitrogen recycling. *Geochim. Cosmochim. Acta* **351**, 96–107 (2023).
19. Breiman, L. Random Forests. *Mach. Learn.* **45**, 5–32 (2001).
20. Geurts, P., Ernst, D. & Wehenkel, L. Extremely randomized trees. *Mach. Learn.* **63**, 3–42 (2006).
21. Chen, T. & Guestrin, C. XGBoost: A Scalable Tree Boosting System. in *Proceedings of the 22nd ACM SIGKDD International Conference on Knowledge Discovery and Data Mining* 785–794 (Association for Computing Machinery, New York, NY, USA, 2016).
22. Petrelli, M., Caricchi, L. & Perugini, D. Machine Learning Thermo-Barometry: Application to Clinopyroxene-Bearing Magmas. *J. Geophys. Res.: Solid Earth* **125**, e2020JB020130 (2020).
23. ZhangZhou, J. *et al.* Predicting sulfide precipitation in magma oceans on Earth, Mars and the Moon using machine learning. *Geochim. Cosmochim. Acta* **366**, 237–249 (2023).
24. Huang, W. H. *et al.* Estimating ferric iron content in clinopyroxene using machine

learning models. *Am. Mineral.* **107**, 1886–1900 (2022).

25. Lei, J., Sen, S., Li, Y. & ZhangZhou, J. Carbon in the deep upper mantle and transition zone under reduced conditions: Insights from high-pressure experiments and machine learning models. *Geochim. Cosmochim. Acta* **332**, 88–102 (2022).
26. Breiman, L. Bagging predictors. *Mach. Learn.* **24**, 123–140 (1996).
27. Ho, T. K. The random subspace method for constructing decision forests. *IEEE Trans. Pattern Anal. Mach. Intell* **20**, 832–844 (1998).
28. Friedman, J. H. Greedy function approximation: A gradient boosting machine. *Ann. Stat.* **29**, 1189–1232 (2001).
29. Syracuse, E. M., van Keken, P. E. & Abers, G. A. The global range of subduction zone thermal models. *Phys. Earth Planet. Inter.* **183**, 73–90 (2010).
30. Schmidt, M. W. & Poli, S. Devolatilization During Subduction. in *Treatise on Geochemistry* 669–701 (Elsevier, 2014).
31. Kupriyanov, I. N. *et al.* Nitrogen fractionation in mica metapelite under hot subduction conditions: Implications for nitrogen ingassing to the mantle. *Chem. Geol.* **628**, 121476 (2023).
32. Sokol, A. G. *et al.* Nitrogen storage capacity of phengitic muscovite and K-cymrite under the conditions of hot subduction and ultra high pressure metamorphism. *Geochim. Cosmochim. Acta* **355**, 89–109 (2023).
