# Supplementary material for: Inefficient nitrogen transport to the lower mantle by sediment subduction
Source: Nat Commun. 2024 Aug 14;15:6998. doi: 10.1038/s41467-024-51524-1 (PMC11324759; doi:10.1038/s41467-024-51524-1)
Supplement: Supplementary file 3 — Description Of Additional Supplementary File [file 41467_2024_51524_MOESM3_ESM.pdf]

Description of Additional supplementary file

Supplementary Data 1

Description: Chemical compositions, phase proportions, and Raman spectra of different phases.

Supplementary Data 2

Description: Calculations of nitrogen preservations and nitrogen deep subduction efficiencies.

Supplementary Data 3

Description: Datasets for machine learning models.
